# Human CXCR5^+^PD‐1^+^ CD8 T cells in healthy individuals and patients with hematologic malignancies

**DOI:** 10.1002/eji.202048761

**Published:** 2020-11-23

**Authors:** Tom Hofland, Anne W.J. Martens, Jaco A.C. van Bruggen, Renate de Boer, Sjoerd Schetters, Ester B.M. Remmerswaal, Frederike J. Bemelman, Mark‐David Levin, Adriaan D. Bins, Eric Eldering, Arnon P. Kater, Sanne H. Tonino

**Affiliations:** ^1^ Department of Hematology, Cancer Center Amsterdam, Amsterdam UMC University of Amsterdam Amsterdam The Netherlands; ^2^ Department of Experimental Immunology, Amsterdam Infection & Immunity Institute, Amsterdam UMC University of Amsterdam Amsterdam The Netherlands; ^3^ Department of Molecular Cell Biology and Immunology Amsterdam UMC Amsterdam The Netherlands; ^4^ Renal Transplant Unit, Amsterdam UMC University of Amsterdam Amsterdam The Netherlands; ^5^ Department of Internal Medicine Albert Schweitzer Hospital Dordrecht The Netherlands; ^6^ Department of Oncology, Amsterdam UMC University of Amsterdam Amsterdam The Netherlands; ^7^ Lymphoma and Myeloma Center Amsterdam LYMMCARE Amsterdam The Netherlands

**Keywords:** CD8 T cells, CXCR5, immune checkpoint blockade, PD‐1 immunotherapy

## Abstract

Immune checkpoint blockade (ICB) has revolutionized cancer therapy, but varying response rates illustrate the need for biomarkers of response. Studies in mice have identified a subset of CD8 T cells that is essential for response to PD‐1 ICB. These CD8 T cells co‐express CXCR5, PD‐1 and Tcf1, and provide effector T cells upon PD‐1 ICB. It is unknown whether similar T cells play a role in PD‐1 ICB in humans.

We studied human peripheral blood and lymph nodes (LNs) for the frequency, phenotype, and functionality of CXCR5^+^PD‐1^+^ CD8 T cells. We find that CXCR5^+^PD‐1^+^ CD8 T cells are memory‐like cells, express Tcf1, and lack expression of effector molecules. CXCR5^+^PD‐1^+^ CD8 T cells produce cytokines upon stimulation, but have limited proliferative capacity. We studied patients with hematologic malignancies with varying response rates to PD‐1 ICB. Specifically in chronic lymphocytic leukemia, in which PD‐1 ICB does not induce clinical responses, CXCR5^+^PD‐1^+^ CD8 T cells show loss of the memory phenotype and increased effector differentiation.

In conclusion, we identified CXCR5^+^PD‐1^+^ CD8 T cells in human peripheral blood and LN, which could play a similar role during PD‐1 ICB. Future studies should analyze CXCR5^+^PD‐1^+^ CD8 T cells during PD‐1 ICB and their importance for therapeutic response.

## Graphical abstract

CXCR5^+^PD‐1^+^ CD8 T cells respond to PD‐1 immune checkpoint blockade in mice. In healthy individuals, human CXCR5^+^PD‐1^+^ CD8 T cells have a similar memory phenotype and functionality. In CLL patients, where PD‐1 blockade does not induce therapeutic responses, CXCR5^+^PD‐1^+^ CD8 T cells show increased differentiation and altered functionality.

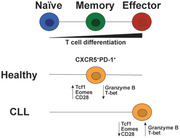

## Introduction

CD8 T cells chronically exposed to antigen develop a state of reduced functionality, a process termed “T‐cell exhaustion” [1]. T‐cell exhaustion is now recognized as an epigenetic and transcriptomic differentiation state distinct from memory and effector T‐cell populations, and is characterized by the upregulation of multiple inhibitory receptors on the cell surface, such as PD‐1 and CTLA‐4^11^. Exhausted T cells progressively lose effector functions, like the ability to produce cytokines, proliferate, and kill target cells, which hampers their ability to control chronic viral infections and tumors [1].

Since T‐cell exhaustion impedes antitumor responses and leads to loss of immune surveillance, reverting T‐cell exhaustion has become a major goal for immunotherapy of cancer. Immune checkpoint blockade (ICB) has changed the landscape of cancer therapy in recent years. ICB therapy aims to rejuvenate antitumor T‐cell responses via the use of mAbs that block signaling through inhibitory receptors on effector T cells. Loss of inhibitory signaling shifts the balance within the tumor microenvironment from an immune‐suppressed environment to a milieu in which effective antitumor responses can be sustained. ICB results in remarkable clinical responses in multiple tumor settings, including melanoma, nonsmall cell lung cancer, and Hodgkin's lymphoma (HL) [2, 3, 4, 5, 6]. However, response rates vary greatly between, and even within, different tumor types. At present, it is unclear what determines whether patients will respond to this therapy, and there is a growing need to identify biomarkers of response.

In recent years, several studies in mice have demonstrated that a specific subset of CD8 T cells is essential for response to PD‐1 ICB [7, 8, 9, 10]. These progenitor cells of exhausted CD8 T cells co‐express PD‐1 with the chemokine receptor CXCR5 and home to lymph node (LN) tissue [7, 9, 10]. CXCR5^+^PD‐1^+^ CD8 T cells were shown to be memory‐like cells with high expression of the transcription factor Tcf1, but in contrast to “classical” exhausted PD‐1^+^ effector T cells, they retain their full functional capabilities [7, 8, 9, 10]. After PD‐1 ICB, CXCR5^+^PD‐1^+^ CD8 T cells were shown to be responsible for the proliferative burst of new effector CD8 T cells and increased control of chronic viral infections and tumors [7, 8, 9, 10]. It is currently unclear whether CXCR5^+^PD‐1^+^ CD8 T cells play a similar important role in responses to ICB in the human setting.

To determine whether CXCR5^+^PD‐1^+^ CD8 T cells play a similar role in response to PD‐1 ICB in the human setting, we studied these cells in healthy individuals and in patients with hematologic malignancies with varying response rates to PD‐1 ICB. In addition to the impressive responses in HL, PD‐1 ICB induces clinical responses in several non‐Hodgkin's lymphomas (NHL) like diffuse large B‐cell lymphoma (DLBCL), multiple myeloma (MM) and follicular lymphoma (FL) patients [11]. In contrast, PD‐1 ICB does not lead to clinical responses in chronic lymphocytic leukemia (CLL) patients, despite the promising effects in mouse models of CLL [11, 12, 13]. Since CLL is characterized by an acquired T‐cell dysfunction, CXCR5^+^PD‐1^+^ CD8 T cells may also be functionally impaired in CLL, which could be an explanation for reduced response rate to PD‐1 ICB [14, 15, 16, 17].

By comparing CXCR5^+^PD‐1^+^ CD8 T cells from healthy individuals and patients with varying hematologic tumors, we aim to determine whether this population also has a pivotal role for response rates to PD‐1 ICB in the human setting.

## Results

### CXCR5^+^PD‐1^+^ CD8 T cells are memory‐like cells that enrich in B‐cell follicles

To study the frequency, phenotype, and localization of CXCR5^+^PD‐1^+^ CD8 T cells, we analyzed matched peripheral blood (PB) and LN samples of healthy controls (HCs) with flow cytometry.

**Figure 1:**
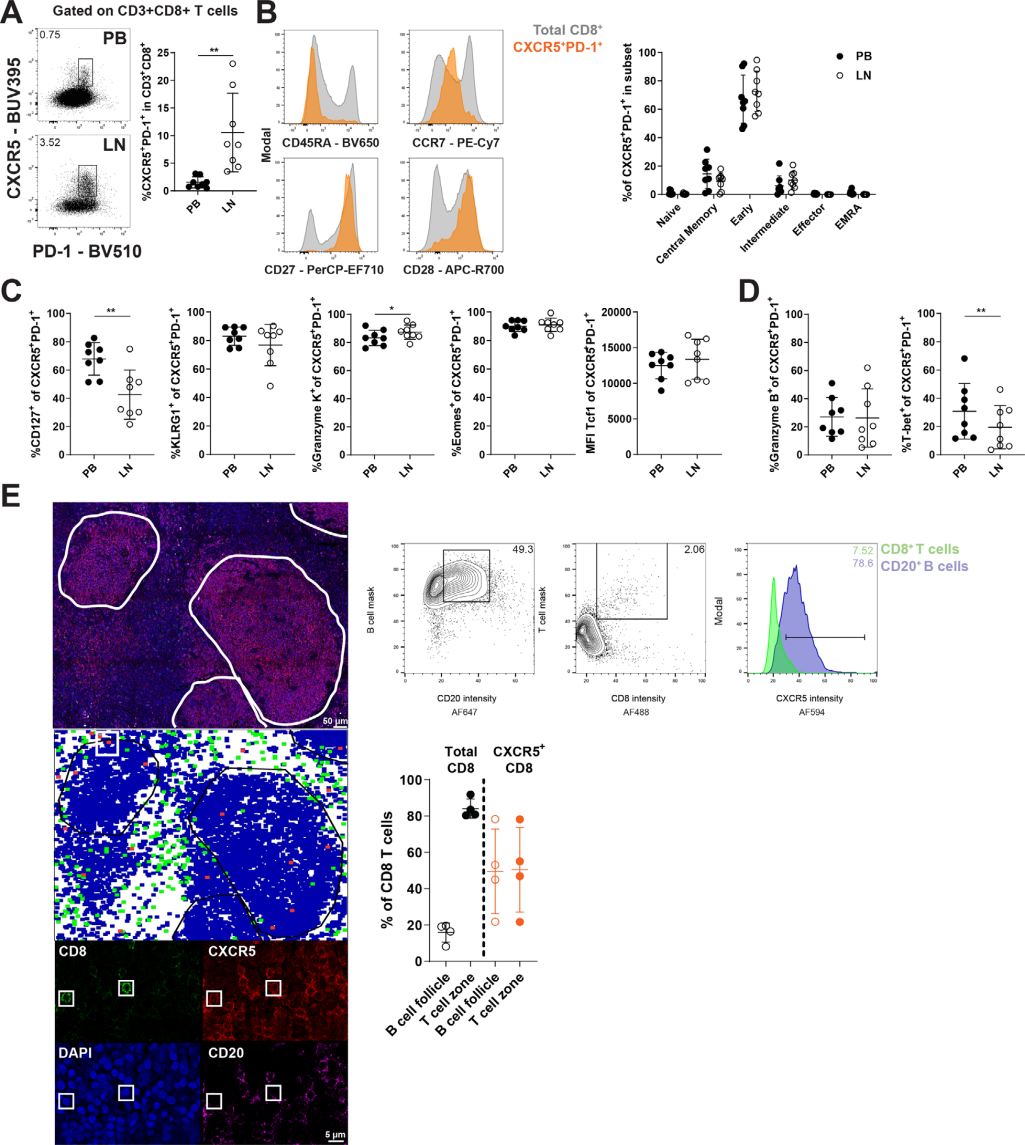
Phenotype and localization of CXCR5^+^PD‐1^+^ CD8 T cells in PB and LN. (A‐D) HC PB and LN samples (*n* = 8, pooled from four independent experiments) were analyzed by flow cytometry to study the frequency and phenotype of CXCR5^+^PD‐1^+^ CD8 T cells. (A) Gating strategy to identify CXCR5^+^PD‐1^+^ within total CD8 T cells and frequency in PB and LN. (B) Differentiation state of CXCR5^+^PD‐1^+^ CD8 T cells based on CD45RA, CCR7, CD27, and CD28. T‐cell subsets are defined as naïve (CD45RA^+^CCR7^+^CD27^+^CD28^+^), central memory (CD45RA^−^CCR7^+^CD27^+^CD28^+^), early effector memory (CD45RA^−^CCR7^−^CD27^+^CD28^+^), intermediate effector (CD45RA^−^CCR7^−^CD27^+^CD28^−^), effector (CD45RA^−^CCR7^−^CD27^−^CD28^−^), and effector memory expressing RA (EMRA; CD45RA^+^CCR7^−^CD27^−^CD28^−^). (C) Expression of molecules associated with memory T‐cell populations in CXCR5^+^PD‐1^+^ CD8 T cells: CD127, KLRG1, granzyme K, Eomes, and Tcf1. (D) Expression of molecules associated with effector T cells in CXCR5^+^PD‐1^+^ CD8 T cells: granzyme B and T‐bet. (E) Example of histo‐cytometry analysis of human lymph nodes. Top panel shows staining with DAPI (blue), CD8 (green), CD20 (magenta), and CXCR5 (red), scale bar = 50 μm (top), 5 μm (bottom) and 40× and 300× magnification, respectively. Fluorescence intensities of cellular surfaces were used to identify B cells (events with high scores in B‐cell mask and CD20 intensity) and CD8 T cells (events with high scores in T‐cell mask and CD8 intensity, also see Materials and Methods section). CXCR5^high^ events were gated in both B‐ and T‐cell populations. Middle panel shows an example of regional distribution of identified B cells (blue), CD8 T cells (green), and CXCR5^high^ CD8 T cells (red). B‐cell follicles were gated to determine cellular localization. The bottom panel shows examples of CXCR5^high^ CD8 T‐cell events (CD8 in green, CXCR5 in red, DAPI in blue, and CD20 in magenta). Finally, graph that depicts the percentage of total CD8 T cells and CXCR5^high^ CD8 T cells in T‐cells zones and B‐cell follicles (*n* = 4 donors, two independent experiments). Data are presented as mean ± SD. *p < 0.05; **p < 0.01 (paired *t*‐test or two‐way ANOVA followed by Sidak's multiple comparisons test)

CXCR5^+^PD‐1^+^ CD8 T cells were identified in both PB and LN, with a significantly higher frequency in LN (Fig. 1A, gating strategy in Supporting Information Fig. S1A). CXCR5^+^PD‐1^+^ CD8 T cells in both PB and LN express high levels of co‐stimulatory molecules CD27 and CD28, and low expression of CCR7, compatible with an early effector memory phenotype (Fig. 1B). The memory‐like phenotype is supported further by the high expression of several markers associated with memory T cells, such as CD127, KLRG1, granzyme K, and eomes (Fig. 1C). CXCR5^+^PD‐1^+^ CD8 T cells have high expression of the transcription factor Tcf1, which in mice is essential for their maintenance and longevity (Fig. 1C) [8]. In line with their subset classification, markers that are upregulated by effector cells, such as T‐bet and granzyme B, are only expressed at low levels by CXCR5^+^PD‐1^+^ CD8 T cells (Fig. 1D).

We determined the localization of CXCR5^+^PD‐1^+^ CD8 T cells in human LN tissue by histo‐cytometry analysis [18]. CD8^+^ T cells mostly localize around CD20^+^ B‐cell follicles in T‐cell zones (Fig. 1E). In contrast, CXCR5^+^ CD8 T cells enrich within B‐cell follicles, indicating a functional role for the CXCR5 chemokine receptor for migration via CXCL13, which was also described by others (Fig. 1E) [7, 10].

In conclusion, CXCR5^+^PD‐1^+^ CD8 T cells are memory‐like cells, which are enriched in LN and localize to B‐cell follicles. These data correspond with results described in mice, suggesting a similar phenotype and role for these cells in humans.

### CXCR5^+^PD‐1^+^ CD8 T cells readily produce cytokines, but have limited proliferative potential

CXCR5^+^PD‐1^+^ CD8 T cells in mice are progenitor cells of exhausted T cells with full functional capacity, in contrast to their exhausted progeny. To determine whether this also applies to human CXCR5^+^PD‐1^+^ CD8 T cells, we stimulated PBMC with PMA/ionomycin and studied cytokine production in relation to other T‐cell subsets (gating strategy in Supporting Information Fig. S1B).

**Figure 2:**
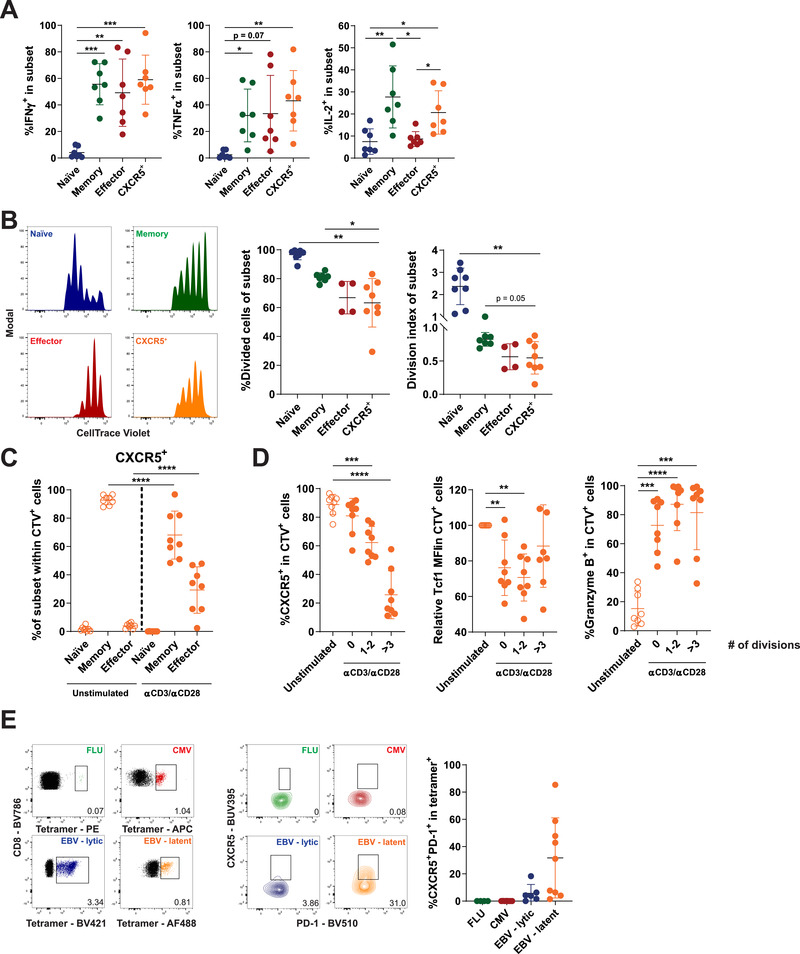
Functionality of CXCR5^+^PD‐1^+^ CD8 T cells after stimulation. (A) HC PBMC fractions were stimulated with PMA/Ionomycin for 5 h, and production of effector cytokines IFN‐γ, TNF‐α, and IL‐2 by CXCR5^+^PD‐1^+^ CD8 T cells and classical T‐cell subsets was analyzed by flow cytometry (*n* = 7, pooled from three independent experiments). (B–D) CXCR5^+^PD‐1^+^ CD8 T cells and T‐cell subsets were sorted and stimulated with anti‐CD3 and anti‐CD28 antibodies for 5 days and analyzed by flow cytometry (*n* = 8, pooled from three independent experiments). (B) Proliferation of T‐cell populations analyzed by dilution of CTV proliferation dye, shown as percentage of divided cells and division index. (C) Differentiation state of sorted CXCR5^+^PD‐1^+^ CD8 T cells before (open circles) and after stimulation for 5 days (filled circles). (D) Expression of CXCR5, Tcf1, and granzyme B by CXCR5^+^PD‐1^+^ CD8 T cells after 5 days, without stimulation (open circles) or after 5 days of stimulation split into cell fractions based on the number of divisions (closed circles). (E) Frequency of CXCR5^+^PD‐1^+^ CD8 T cells within virus‐specific CD8 T cells toward influenza (FLU), cytomegalovirus (CMV), and Epstein–Barr virus (EBV) (*n* = 8, pooled from four independent experiments). T‐cell subsets in all panels are defined as naïve (CD45RA^+^CD27^+^), memory (CD45RA^−^CD27^+^), and effector (CD45RA^+/−^CD27^−^) T cells. Data are presented as mean ± SD. *p < 0.05; **p < 0.01; ***p < 0.001, ****p < 0.0001 (repeated measures one‐way ANOVA).

After stimulation with PMA/ionomycin, antigen‐experienced memory and effector T cells produce effector cytokines such as IFN‐γ, TNF‐α, or IL‐2, in contrast to naïve T cells (Fig. 2A). CXCR5^+^PD‐1^+^ CD8 T cells also readily produce cytokines, and are able to quickly produce IL‐2 similar to memory CD8 T cells, which sets CXCR5^+^PD‐1^+^ CD8 T cells apart from effector CD8 T cells (Fig. 2A).

In mice, the primary function of these cells is not to produce cytokines, but to proliferate and sustain the effector T‐cell pool. To determine the proliferative potential of CXCR5^+^PD‐1^+^ CD8 T cells, we sorted CXCR5^+^, naïve, memory, and effector CD8 T cells and stimulated these cells with anti‐CD3 and anti‐CD28 antibodies for 5 days. As expected, naïve CD8 T cells showed the highest rate of proliferation (measured as the frequency of divided cells and division index), and we observe a gradual decrease in proliferative potential in memory and effector pools (Fig. 2B). Despite their memory‐like phenotype and cytokine production profile, CXCR5^+^ CD8 T cells showed limited proliferation, at the same level as effector T cells (Fig. 2B).

Sorted CXCR5^+^ CD8 T cells differentiated into effector‐like cells after activation, to a similar extent as sorted memory cells (Fig. 2C and Supporting Information Fig. S2A–D). In line with their differentiation to an effector subset, CXCR5 and Tcf1 expression gradually decreased after multiple divisions, while the expression of granzyme B increased (Fig. 2D).

The level of proliferation of CXCR5^+^PD‐1^+^ CD8 T cells is lower than expected for memory‐like cells. An explanation for their limited proliferative potential could be that CXCR5^+^PD‐1^+^ CD8 T cells develop selectively to chronic antigen stimuli, as is the case in mice. To determine which type of antigens induce CXCR5^+^PD‐1^+^ CD8 T cells, we used tetramers to study antigen‐specific CD8 T cells toward influenza (FLU), cytomegalovirus (CMV) or Epstein–Barr virus (EBV) by flow cytometry. As expected, the CD8 T‐cell pool specific for FLU, an acute infection, does not contain CXCR5^+^PD‐1^+^ CD8 T cells (Fig. 2E). Although we could not detect CXCR5^+^PD1^+^ CD8 T cells directed toward the chronic infection CMV, we did identify these cells directed against EBV, especially to epitopes derived from proteins of the latency phase (Fig. 2E).

Taken together, these results indicate that CXCR5^+^ CD8 T cells readily produce effector cytokines, but have limited proliferative potential compared to central memory T cells. The limited proliferation of CXCR5^+^ CD8 T cells may be explained by the fact that these cells mainly develop toward chronic antigens and have over time divided more than central memory T cells due to persistent antigen exposure.

### CXCR5^+^PD‐1^+^ CD8 T cells show increased effector differentiation in CLL, but not other NHL

In order to analyze the clinical relevance of our results, we analyzed CXCR5^+^PD1^+^ CD8 T cells in patients with hematologic malignancies with varying response rates to PD‐1 ICB. In CLL, PD‐1 ICB therapy shows disappointing results, with no objective responses in patients unless they develop Richter's transformation [13]. Other NHL types show better response rates to PD‐1 ICB, such as DLBCL, MM, and FL patients, while HL shows the highest response rate to PD‐1 ICB [4, 11]. Since it is established that T cells in CLL patients acquire several phenotypic and functional alterations [14, 15, 16], we analyzed CXCR5^+^PD‐1^+^ CD8 T cells across these malignancies to determine whether there is a relation between these cells and the response to PD‐1 ICB.

**Figure 3:**
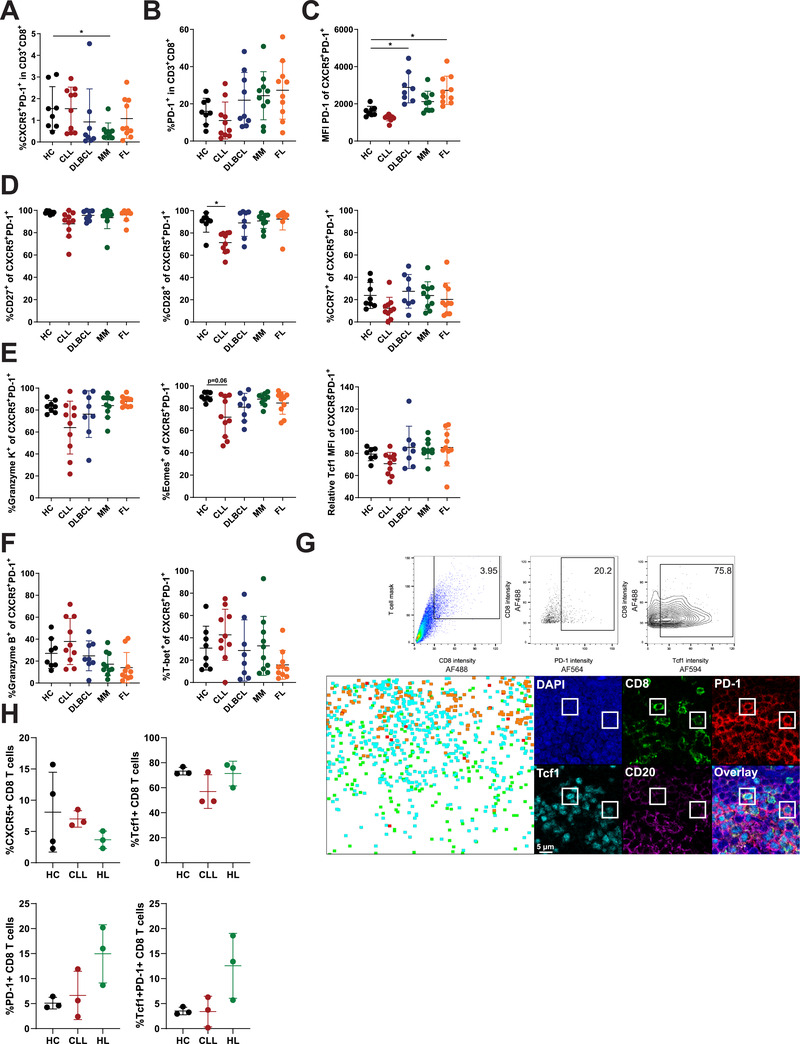
Increased effector differentiation of CXCR5^+^PD‐1^+^ CD8 T cells in CLL. (A–F) Flow cytometry analysis pooled from ten independent experiments of PBMC from HC (*n* = 8), CLL patients (*n* = 10), DLBCL (*n* = 8), MM (*n* = 10), and FL (*n* = 10). (A) Frequency of CXCR5^+^PD‐1^+^ within CD8 T cells in PB. (B) Frequency of PD‐1^+^ CD8 T cells within total CD8 in PB. (C) Expression level of PD‐1 by CXCR5^+^PD‐1^+^ CD8 T cells. (D) Expression of CD27, CD28, and CCR7 in CXCR5^+^PD‐1^+^ CD8 T cells. (E) Expression of molecules associated with memory T cells by CXCR5^+^PD‐1^+^ CD8 T cells. (F) Expression of molecules associated with effector T cells by CXCR5^+^PD‐1^+^ CD8 T cells. (G) Example of gating strategy used to define PD‐1^+^ and Tcf1^+^ CD8 T cells in LN tissues for histo‐cytometry analysis. Top panels from left to right show gating of CD8 T cells, PD‐1^+^ CD8 T cells, and Tcf1^+^ CD8 T cells. Bottom row shows regional distribution of PD‐1^−^Tcf1^−^ CD8 T cells (green), PD‐1^−^Tcf‐1^+^ CD8 T cells (cyan), PD‐1^+^Tcf1^−^ CD8 T cells (red), and PD‐1^+^Tcf1^+^ CD8 T cells (orange) in HL‐derived LN tissue, and an example of two PD‐1+Tcf1+ CD8 T cells (DAPI in blue, CD8 in green, PD‐1 in red, Tcf1 in cyan, and CD20 in magenta), scale bar = 5 μm and 300× magnification. (H) Frequency of CXCR5+ CD8 T cells, PD‐1^−^Tcf‐1^+^ CD8 T cells, PD‐1^+^Tcf1^−^ CD8 T cells, and PD‐1^+^Tcf1^+^ CD8 T cells in HC‐ (*n* = 4), CLL‐ (*n* = 3), or HL‐derived (*n* = 3) LN tissue (pooled from three independent experiments). Data are presented as mean ± SD. *p < 0.05 (Kruskal–Wallis test followed by Dunn's multiple comparisons test).

The frequency of CXCR5^+^PD‐1^+^ CD8 T cells in PB is low in all malignancies, and is not changed dramatically across different NHL regardless of response rates to PD‐1 ICB (Fig. 3A). The frequency of CXCR5^+^PD‐1^+^ CD8 T cells does not correlate with the amount of PD‐1^+^ CD8 T cells in PBMC (Fig. 3B). Interestingly, the expression of PD‐1 per cell was lower on CXCR5^+^PD‐1^+^ CD8 T cells in CLL compared to HC and other NHL (Fig. 3C). In CLL, CXCR5^+^PD‐1^+^ CD8 T cells show increased differentiation, whereas in DLBCL, MM, and FL, CXCR5^+^PD‐1^+^ CD8 T cells remain in a memory‐like state similar to HC, with high expression of CD27, CD28, and CCR7 (Fig. 3D and Supporting Information Fig. S3). The loss of a memory phenotype was supported by reduced expression of the memory‐associated proteins granzyme K, eomes, and Tcf1 in CXCR5^+^PD‐1^+^ CD8 T cells in CLL (Fig. 3E). In contrast, effector molecules granzyme B and T‐bet showed a trend toward a higher expression in CLL (Fig. 3F).

As the presence of CXCR5^+^PD‐1^+^ CD8 T cells at the tumor site may be important in order to respond to PD‐1 ICB, we analyzed CLL LN biopsies for the presence of CXCR5^+^ CD8 T cells by histo‐cytometry analysis. We performed an additional staining to identify PD‐1^+^Tcf1^+^ CD8 T cells (Fig. 3G), whose frequency positively correlates with response to PD ICB in melanoma [19, 20, 21]. Although these studies did not always include CXCR5 in their gating analysis, extensive phenotyping of PD‐1^+^Tcf1^+^ T cells in solid tumors showed CXCR5 expression on a subset of these cells, and also many functional similarities between these cells and the CXCR5^+^ CD8 T cells we studied here [19, 20, 21]. We compared CLL‐derived LN tissues to HC LN (also see Fig. 1), and also included LN tissues from HL patients, of whom almost 90% respond to PD‐1 ICB, as a control for hematologic tumors that do respond [4]. We find no difference in the frequency of CXCR5^+^ CD8 T cells among HC, CLL, or HL LN tissues (Fig. 3H). CD8 T cells in all LN tissues frequently express Tcf1, which is probably due to the high frequency of naïve and memory T cells in LN (Fig. 3H and Supporting Information Fig. S3B). In line with the high response rates to PD‐1 ICB, HL‐derived LN tend to have an increased frequency of both exhausted PD‐1^+^ CD8 T cells and the progenitor PD‐1^+^Tcf1^+^ CD8 T cells compared to HC and CLL LN tissues (Fig. 3H).

In conclusion, we observe increased differentiation of CXCR5^+^PD‐1^+^ CD8 T cells combined with a lower expression of PD‐1 in CLL, but not in other NHL with better response rates to PD‐1 ICB. Although we did not find an altered frequency of CXCR5^+^ CD8 T cells in CLL, the frequency of PD‐1^+^Tcf1^+^ CD8 T cells in CLL LN appears to be lower than HL LN.

### Altered functionality of CXCR5^+^PD‐1^+^ CD8 T cells in CLL upon stimulation

To study whether the altered phenotype of CXCR5^+^PD‐1^+^ CD8 T cells in CLL patients also leads to functional changes, we stimulated CXCR5^+^PD‐1^+^ CD8 T cells from CLL patients to study cytokine production, proliferation, and differentiation.

**Figure 4:**
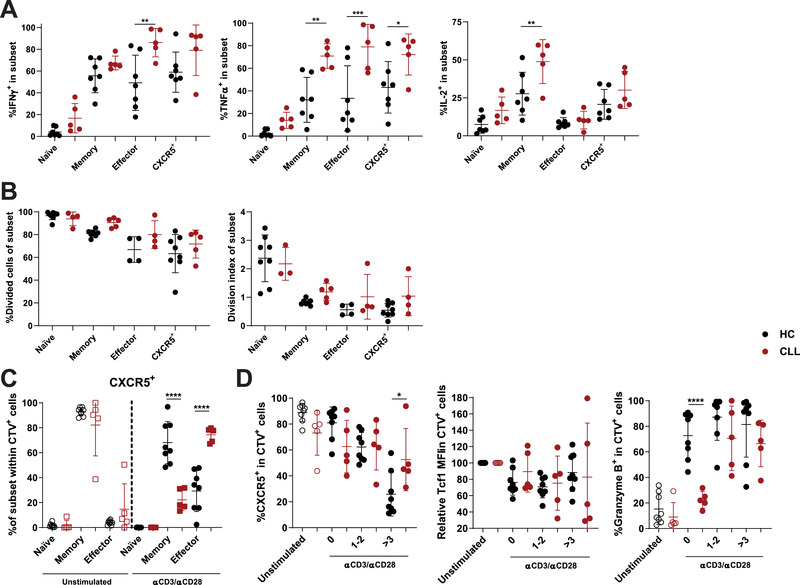
Functionality of CXCR5^+^PD‐1^+^ CD8 T cells in CLL. (A) IFN‐γ, TNF‐α, and IL‐2 production by T‐cell subsets after PMA/ionomycin stimulation for 5 h (HC: *n* = 7, CLL: *n* = 5, pooled from three independent experiments). (B) Proliferation of sorted T‐cell subsets from HC or CLL after stimulation with anti‐CD3 and anti‐CD28 for 5 days (HC: *n* = 7; CLL: *n* = 5, pooled from four independent experiments). (C) Differentiation state of CXCR5^+^PD‐1^+^ CD8 T cells from HC or CLL before (open symbols) and after stimulation for 5 days (closed symbols) (HC: *n* = 7; CLL: *n* = 5, pooled from four independent experiments). (D) Expression of CXCR5, Tcf1, and granzyme B by CXCR5^+^PD‐1^+^ CD8 T cells of HC or CLL before (open symbols) and after stimulation for 5 days (closed symbols) (HC: *n* = 7; CLL: *n* = 5, pooled from four independent experiments). T‐cell subsets are defined as naïve (CD45RA^+^CD27^+^), memory (CD45RA^−^CD27^+^), and effector (CD45RA^+/−^CD27^−^) T cells. Data are presented as mean ± SD and measured by flow cytometry. *p < 0.05; **p < 0.01; ***p < 0.001, ****p < 0.0001 (two‐way ANOVA followed by Sidak's multiple comparisons test).

Earlier reports showed increased IFN‐γ and TNF‐α production by CD8 T cells of CLL patients upon stimulation [16]. In line with increased differentiation, CXCR5^+^PD‐1^+^ CD8 T cells of CLL patients produced higher levels of IFN‐γ and TNF‐α compared to HC (Fig. 4A). Interestingly, CD8 T cells in CLL show elevated production of IFN‐γ and TNF‐α across T‐cell subsets, indicating that increased production of these cytokines in CLL is not caused merely by skewed differentiation of the global CD8 T‐cell compartment (Fig. 4A). The proliferative capacity of CXCR5^+^PD‐1^+^ CD8 T cells in CLL was comparable to effector T‐cell populations and not different from HC (Fig. 4B). However, after 5 days of stimulation, significantly more CXCR5^+^PD‐1^+^ CD8 T cells differentiated into effector CD8 T cells in CLL (Fig. 4C). Despite this increased differentiation, the loss of CXCR5 and upregulation of granzyme B seemed less profound in CXCR5^+^PD‐1^+^ CD8 T cells in CLL after stimulation, which could indicate an altered ability of CXCR5^+^PD‐1^+^ CD8 T cells to respond to stimulation in the CLL microenvironment (Fig. 4D).

In conclusion, CXCR5^+^PD‐1^+^ CD8 T cells in CLL patients show increased effector differentiation and function directly ex vivo and after stimulation.

## Discussion

The clinical responses induced by ICB in specific cancer types have fueled extensive clinical testing of these therapies in many malignancies. However, due to great variation in response rates between and within tumor types, there is an urgent need for biomarkers of clinical response. CXCR5^+^PD‐1^+^ CD8 T cells were identified in mice as the essential T‐cell subset for immunological response to PD‐1 ICB [7, 8, 9, 10]. To determine whether CXCR5^+^PD‐1^+^ CD8 T cells could play a similar important role in humans, we studied the localization, phenotype, and functionality of CXCR5^+^PD‐1^+^ CD8 T cells in HC and patients with hematologic malignancies with varying responses to PD‐1 ICB therapy. We found that CXCR5^+^PD‐1^+^ CD8 T cells in HC are memory‐like T cells with low expression of effector molecules that adequately produce effector cytokines and differentiate into effector cells upon stimulation. In CLL, where PD‐1 ICB does not induce clinical responses, CXCR5^+^PD‐1^+^ CD8 T cells show increased differentiation ex vivo, and altered differentiation toward effector populations after stimulation, indicating that functional modulation by the CLL microenvironment could hamper responses to PD‐1 ICB. Indeed, several of the observed alterations in CLL are not present in patients with other hematologic malignancies with better response rates to PD‐1 ICB.

Our results on the phenotype and function of human CXCR5^+^PD‐1^+^ CD8 T cells largely corroborate data from mouse studies [7, 8, 9, 10]. Human CXCR5^+^PD‐1^+^ CD8 T cells have a similar memory‐like phenotype and express the transcription factor Tcf1, which in mice is essential for the maintenance of the population [8]. Upon stimulation, CXCR5^+^PD‐1^+^ CD8 T cells proliferate and give rise to effector T‐cell populations. Although the proliferation rate of CXCR5^+^PD‐1^+^ CD8 T cells is quite low in our experiments, it could be that proliferation of these cells is amplified by PD‐1 ICB, as is the case in mice [8, 9, 10]. Finally, we could detect CXCR5^+^PD‐1^+^ CD8 T cells in antigen‐specific T‐cell populations toward chronic viral infections (EBV), but not an acute viral infection (FLU), indicating that chronic antigen stimulation could be required for CXCR5^+^PD‐1^+^ CD8 T cells to develop, as is the case in mice [7, 8, 9, 10].

One aspect that is still under debate is the location of CXCR5^+^PD‐1^+^ CD8 T cells within LN. Conflicting reports in murine LN placed CXCR5^+^PD‐1^+^ CD8 T cells in both T‐cell zones and B‐cell follicles [7, 9, 10]. We find that CXCR5^+^PD‐1^+^ CD8 T cells migrate into B‐cell follicles, in contrast to CXCR5^−^ CD8 T cells. These results are in line with earlier reports that show migration of CXCR5^+^ CD8 T cells via CXCL13, and locate CXCR5^+^ CD8 T cells in B‐cell follicles within tonsil and LN tissue [7, 22]. Conflicting reports on the localization of these cells may reflect the expression of both CXCR5 and CCR7 by CXCR5^+^PD‐1^+^ CD8 T cells, which control migration into B‐cell follicles or T‐cell zones, respectively.

Due to the importance of CXCR5^+^PD‐1^+^ CD8 T cells for response to ICB in mice, several other studies have looked in tumor samples for a human analogue of this progenitor T cell. Multiple reports using flow cytometry and single‐cell sequencing on solid tumors show the presence of memory‐like T cells among tumor infiltrating lymphocytes (TILs) with similarities to CXCR5^+^PD‐1^+^ CD8 T cells in melanoma, lung cancer, pancreatic cancer, and colon cancer [19, 20, 21, 23, 24, 25, 26, 27]. Although CXCR5 is not included in all analyses, the identified subsets display increased T‐cell functionality within tumor infiltrating lymphocytes, and PD‐1^+^Tcf1^+^ CD8 T cells are positively correlated with survival and clinical response to PD‐1 ICB in melanoma [19, 20, 21, 23, 24]. These results support a similar phenotype and role for CXCR5^+^PD‐1^+^ CD8 T cells during PD‐1 ICB in the human setting.

We analyzed the frequency and phenotype of CXCR5^+^PD‐1^+^ CD8 T cells in hematologic malignancies to determine whether they could play a similar important role of therapeutic response in those patients. Particularly in CLL, where there is no objective response to PD‐1 ICB, we observe phenotypical alterations of CXCR5^+^PD‐1^+^ CD8 T cells. PD‐1 expression is expressed at lower levels on these cells, especially compared to other NHL with better response rates, which may indicate that the low expression of the target molecule of ICB is influencing the response to therapy. We also observe increased effector differentiation of CXCR5^+^PD‐1^+^ CD8 T cells in CLL patients, both directly ex vivo and after stimulation, which could hamper the ability of these cells to induce long‐lasting immune responses. Indeed, all identified T‐cell subtypes that correlate with response to ICB in other studies are characterized by memory T‐cell signatures [19, 20, 21, 25]. Loss of this phenotype could therefore explain a low response of CLL patients to ICB therapy. Furthermore, the reduced expression of CD28 by CXCR5^+^PD‐1^+^ CD8 T cells could play an important role in the lack of therapeutic response, as PD‐1 ICB mainly targets CD28 signaling, and rescue of exhausted CD8 T cells is dependent on CD28 expression [28, 29]. Although all these aspects in CXCR5^+^PD‐1^+^ CD8 T cells themselves could play a role, the low levels of tumor neo‐antigens combined with the poor antigen presentation by CLL cells could also play a role for the low response rates to PD‐1 ICB [30, 31].

We did not observe expansion of PD‐1^+^Tcf1^+^ CD8 T cells in CLL‐infiltrated LN tissue, in contrast to LN of HL patients, which show higher response rates to PD‐1 ICB. Similar positive correlations between PD‐1^+^Tcf1^+^ CD8 T cells and response to PD‐1 ICB have been described in solid tumors [19, 20, 21, 25]. What may complicate similar analyses in hematologic malignancies is the LN tissue as a microenvironment, where immune cell infiltrates are different from solid tissues. CD8 T cells in HC LN mainly consist of naïve and memory cells, which all express Tcf1, which is the main discriminator for response to PD‐1 ICB in solid tumors.

Detailed analysis of tumor‐specific CD8 T‐cell responses may help to determine the presence of CXCR5^+^PD‐1^+^Tcf1^+^ CD8 T cells despite a high background of PD‐1^+^Tcf1^+^ memory cells in the same LN tissue. Although we did not have these samples available, tissues and PBMC from patients with hematologic malignancies who received PD‐1 ICB therapy should be analyzed to study the dynamics of CXCR5^+^PD‐1^+^ CD8 T cells to definitely prove association with response to therapy.

In conclusion, we find a similar phenotype and functionality of CXCR5^+^PD‐1^+^ CD8 T cells in human PB and LN as was described in mice, which may indicate a similar pivotal role for the response to PD‐1 ICB. In addition, we demonstrated hampered differentiation properties of these CXCR5^+^PD‐1^+^ CD8 T cells in patients with CLL. Future studies in hematologic malignancies should analyze CXCR5^+^PD‐1^+^ CD8 T cells within tumor‐specific CD8 T‐cell populations during PD‐1 ICB therapy to determine whether CXCR5^+^PD‐1^+^ CD8 T cells are a biomarker for therapeutic response.

## Materials and methods

### Healthy donor and CLL patient samples

HC PBMCs were obtained from buffycoats from Sanquin Blood Supply, Amsterdam. Monoclonal B‐cell lymphocytosis was excluded by CD19, CD5, κ, and λ immunophenotyping. Matched PBMC and LN samples were collected from patients undergoing renal transplantation, as described previously [32]. PBMC samples from untreated CLL, DLBCL, FL, and MM patients were collected at the Albert Schweitzer Hospital in Dordrecht and the Amsterdam University Medical Centers, location AMC in Amsterdam (see Table 1 for patient overview). PBMC were isolated and either used directly or cryopreserved as described earlier [33]. Paraffin‐embedded LN tissue was obtained from the pathology department of the Amsterdam University Medical Centers, location AMC. Reactive LN tissues free of malignancy were defined as HC LN. Ethical approval was provided by the medical ethical committee at the Amsterdam University Medical Centers, location AMC in Amsterdam, and written informed consent was obtained in accordance with the Declaration of Helsinki.

**Table 1:** Patient characteristics

	CLL	DLBCL	FL	MM
Number	10	8	10	10
Age, years, mean (min‐max)	67.7 (40‐84)	60 (40‐77)	59.9 (33‐77)	58.5 (45‐83)
Disease stage (%)	Rai 0 (90%)	Stage I‐A (12.5%)	Stage III‐A (40%)	Stage 1 (30%)
	Rai 1 (10%)	Stage III (25%)	Stage IV‐A (60%)	Stage 2 (20%)
		Stage IV‐A (50%)		Stage 3 (50%)
		Stage IV‐B (12.5%)		

### Phenotypic analysis by flow cytometry

PBMC were washed with ice‐cold PBS containing 0.5% bovine serum albumin (PBA) and stained for 30 min at 4°C with virus tetramers. Afterward, cells were incubated with mAbs for surface staining for 30 min at 4°C. Information on all tetramers and antibodies used in the study can be found in Supporting Information Table S1. For intracellular stainings, cells were washed with PBA and fixed using the FoxP3/Transcription Factor Staining Buffer Set (ThermoFisher, Waltham, MA, USA) according to manufacturer's instructions. Cells were then stained intracellularly with mAbs for 30 min at 4°C. Cells were washed and analyzed on a LSR Fortessa cytometer (BD Biosciences, Franklin Lakes, NJ, USA). Data were analyzed using Flowjo v10 (TreeStar, San Carlos, CA, USA).

### Confocal microscopy and histocytometry

Paraffin‐embedded tissue slides of lymph nodes were used for confocal microscopy and histocytometry analysis. Slides were deparaffinized with xylene and rehydrated with ethanol and PBS. Antigen retrieval was performed at 95°C with Tris‐EDTA for 20 min. Slides were allowed to cool down to room temperature, washed twice with PBS, and blocked with 10% BSA in PBS for 30 min. Slides were incubated overnight with monoclonal primary antibodies at 4°C. Cells were washed twice with PBS, and incubated with secondary antibodies for 30 min at room temperature. Slides were washed with PBS and stained with 4,6‐diamidino‐2‐phenylindole (DAPI, 0,1 μg/mL) for 10 min. Slides were washed and mounted overnight with Fluoromount G (ThermoFisher). Images were acquired with a Leica TCS SP8 X microscope (Leica Microsystems, Wetzlar, Germany). After acquisition, the tiled images were merged and compensated using the LAS X Merge and Channel Dye Separation module, respectively. Next, 3D stacked and compensated images were analyzed using histocytometry in Imaris 7.4 (Bitplane, Belfast, United Kingdom) and FlowJo as previously described [18]. Cellular regions in images were determined by nuclear segmentation with the DAPI stain using the surface creation module in Imaris. Statistics of created surfaces were exported into Excel (Microsoft) and then into Flowjo 10. CD20^+^CD8^−^ B cell and CD20^−^CD8^+^ T cell masks were created in Imaris to support gating of lymphocyte subsets. Regional gates of B‐cell follicles were used to determine localization of lymphocytes within LN tissue.

### Cytokine production assays

Fresh PBMC were left resting overnight and treated with PMA (10 ng/mL) and ionomycin (1 μg/mL), or left untreated for 4 h in the presence of brefeldin A (10 μg/mL; all Sigma‐Aldrich, St. Louis, MO, USA) and GolgiStop (BD Biosciences). Afterward, cells were washed and staining was performed as described above. Fixation and intracellular staining was performed using the Cytofix/Cytoperm Solution kit (BD Biosciences). Cells were analyzed on a LSR Fortessa cytometer (BD Biosciences), and data were analyzed with Flowjo v10.

### Proliferation and differentiation assays

CD8 T cells were enriched from fresh PBMC using Easysep CD8 T negative selection kit (Stemcell Technologies, Vancouver, Canada) according to manufacturer's instructions. Next, cells were stained with surface antibodies for 30 min at room temperature, washed, and sorted using a FACSAria Ilu SORP cellsorter (BD Biosciences). Sorted CD8 subsets are defined as CXCR5^+^CD8^+^ (CXCR5^+^ CD8), CXCR5^−^CD45RA^+^CD27^+^CD8^+^ (naïve), CXCR5^−^CD45RA^−^CD27^+^CD8^+^ (memory), and CXCR5^−^CD45RA^+/−^CD27^−^CD8^+^ (effector). Afterward, sorted fractions were labeled with CellTrace Violet (ThermoFisher) and mixed back separately with unsorted and unlabeled autologous PBMC. Next, all PBMC fractions were stimulated with anti‐CD3 (clone 1XE) and anti‐CD28 (clone 15E8) antibodies and IL‐2 (50 U/mL, Peprotech, London, United Kingdom) for 5 days. Afterward, cells were stained with mAbs as described above.

### Statistics

Data were analyzed using paired Student's *t*‐test, repeated measures one‐way ANOVA with Bonferroni correction, two‐way repeated measures ANOVA with Sidak's multiple comparisons test, or Kruskal–Wallis test followed by Dunn's multiple comparisons test, as indicated. Statistical tests were performed using GraphPad Prism 6. Differences were considered statistically significant when *p*‐values were ≤0.05. Data are presented as mean ± SEM.

## Conflict of interest

The author's declare no commercial or financial conflict of interests.

### Peer review

The peer review history for this article is available at https://publons.com/publon/10.1002/eji.202048761.

**Abbreviations**
CLL: chronic lymphocytic leukemia
DLBCL: diffuse large B‐cell lymphoma
FL: follicular lymphoma
HC: healthy control
HL: Hodgkin's lymphoma
ICB: immune checkpoint blockade
MM: multiple myeloma
NHL: non‐Hodgkin's lymphomas
PB: peripheral blood

## Supporting information

Supporting information

## Data Availability

The data that support the findings of this study are available from the corresponding author upon reasonable request.
