# Supplementary material for: Human CXCR5+PD‐1+ CD8 T cells in healthy individuals and patients with hematologic malignancies
Source: Eur J Immunol. 2020 Nov 23;51(3):703–13. doi: 10.1002/eji.202048761 (PMC7984320; doi:10.1002/eji.202048761)
Supplement: Supplementary file 1 — Supporting information [file EJI-51-703-s001.pdf]

Antibody	Fluorochrome	Clone	Company	Catalogue number
**Flow cytometry**				
CXCR5	BUV395	RF8B2	BD Biosciences	740266
CD3	BUV496	UCHT1	BD Biosciences	564809
PD-1	BV510	EH12.1	BD Biosciences	563076
CD8	BV786	RPA-T8	BD Biosciences	563823
CD27	PerCP-eFluor710	O323	ThermoFisher	46-0279-42
CD45RA	BV650	HI100	BD Biosciences	563963
CCR7	PE-Cy7	3D12	BD Biosciences	557648
CD28	APC-R700	CD28.2	BD Biosciences	565181
CD127	BV650	A019D5	Biolegend	351326
KLRG1	PE/Dazzle594	SA231A2	Biolegend	367710
CX3CR1	PE-Cy7	2A9-1	Biolegend	341612
Tim-3	BV650	7D3	BD Biosciences	565564
CD244	PE/Dazzle594	C1.7	Biolegend	329522
CD160	PE-Cy7	BY55	Biolegend	341211
LAG-3	APC-R700	T47-530	BD Biosciences	565774
c-Kit	BV711	104D2	Biolegend	313230
Ki-67	BV711	Ki-67	Biolegend	350515
Tcf1	Unconjugated	C63D9	Cell Signaling	2203S
Granzyme K	PerCP-eFluor710	G3H69	ThermoFisher	46-8897-41
Granzyme B	AF700	GB11	BD Biosciences	560213
CTLA-4	PerCP-eFluor710	14D3	ThermoFisher	46-1529-42
Eomes	PerCP-eFluor710	WD1928	ThermoFisher	46-4877-42
T-bet	PE-CF594	O4-46	BD Biosciences	562467
Bcl6	PE-Cy7	K112-91	BD Biosciences	563582
Goat anti-rabbit	AF594		ThermoFisher	A-11012
Goat anti-rabbit	AF700		ThermoFisher	A-21038
Fixable Viability Dye	APC-eFluor780		ThermoFisher	65-0865-14
CD107a	PE-Cy7	H4A3	BD Biosciences	561348
IFNγ	BV421	B27	BD Biosciences	562988
IL-2	PE/Dazzle594	MQ1-17H12	Biolegend	500343
TNFα	AF700	MAb11	BD Biosciences	561023
CXCR5	AF647	RF8B2	BD Biosciences	558113
CD8	PE-Cy7	RPA-T8	ThermoFisher	25-0088-42
PD-1	PE-Cy7	EH12.1	BD Biosciences	561272
**Confocal microscopy**				
CXCR5	Unconjugated	D6L3C	Cell Signaling	CST 72172S
CD8	Unconjugated	C8/144B	Biolegend	372902
CD20	Unconjugated	Polyclonal	Abcam	ab194970
PD-1	Unconjugated	EH33	Cell Signaling	43248
Tcf1	Unconjugated	C63D9	Cell Signaling	2203S
Goat anti-rabbit	AF594		ThermoFisher	A-11012
Donkey anti-goat	AF647		ThermoFisher	A-21447
Goat anti-mouse IgG1	AF488		ThermoFisher	A-21121
Goat anti-mouse IgG2a	AF546		ThermoFisher	A-21133

**Tetramers**				
**Influenza A**				
HLA-A2/GILGFVFTL	PE		Sanquin Blood Supply	
**Epstein-Barr virus (EBV)**				
HLA-A2/GLCTLVAML	AF488		NIH Tetramer Core Facility	
HLA-A2/FLYALALLL	BV421		NIH Tetramer Core Facility	
HLA-A2/CLGGLLTMV	BV421		NIH Tetramer Core Facility	
HLA-B7/RPPIFIRRL	AF488		NIH Tetramer Core Facility	
HLA-B7/RLRAEAQVK	BV421		NIH Tetramer Core Facility	
HLA-B8/RAKFKQLL	BV421		NIH Tetramer Core Facility	
HLA-B8/FLRGRAYGL	AF488		NIH Tetramer Core Facility	
**Cytomegalovirus (CMV)**				
HLA-A2/VLEETSVML	APC		Sanquin Blood Supply	
HLA-A2/NLVPVATV	APC		Sanquin Blood Supply	
HLA-B7/TPRVTGGGAM	APC		Sanquin Blood Supply	
HLA-B8/QIKVRVDMV	APC		Sanquin Blood Supply	
HLA-B8/ELRRKMMYM	APC		Sanquin Blood Supply	
HLA-B8/ELKRKMIYM	APC		Sanquin Blood Supply	

Supplemental Figure 1 - Gating strategy of CXCR5+PD-1+ CD8 T cells

A

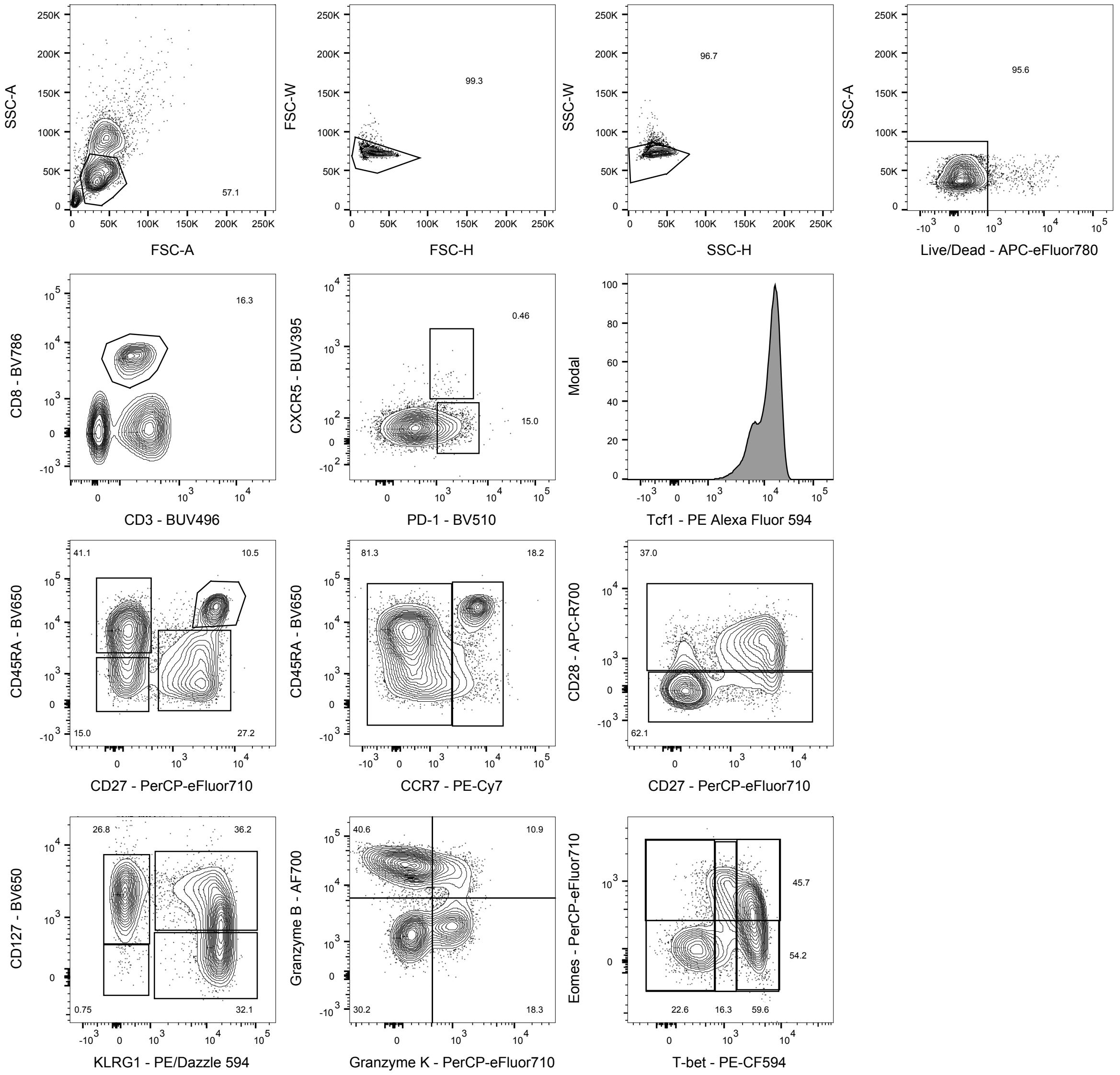

B

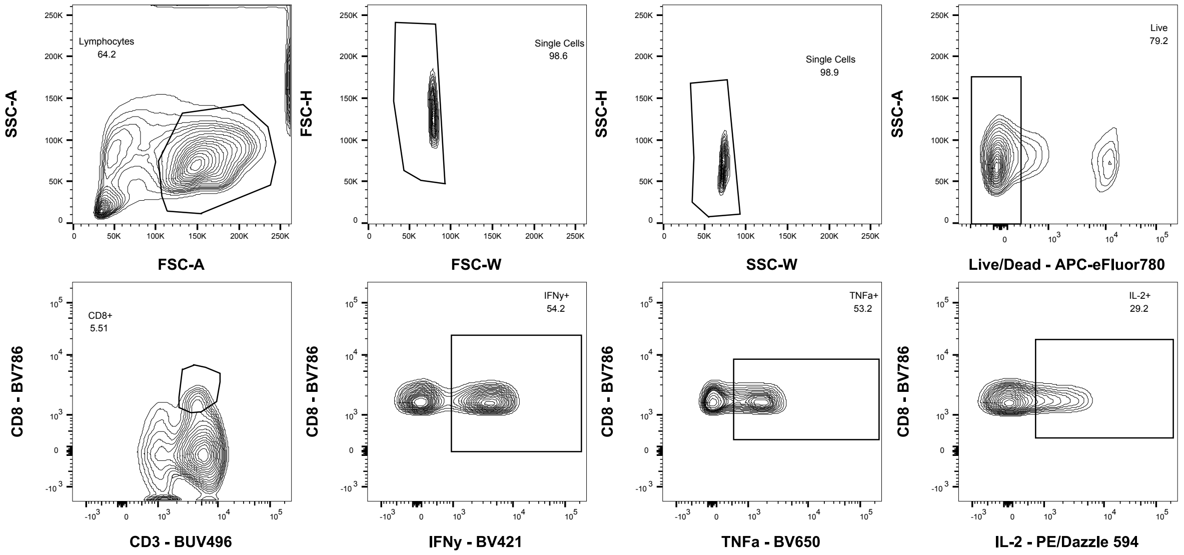

Supplemental Figure 1. Gating strategy for CXCR5$^+$PD-1$^+$ CD8 T cells.

(A) Gating strategy for CXCR5$^+$PD-1$^+$ CD8 T cells and other phenotypic markers. Top row from left to right: gating of lymphocytes, single cells (two panels) and live cells. Second row from left to right: gating of CD8 T cells out of live cells, gating of CXCR5$^+$PD-1$^+$ CD8 T cells and expression of Tcf1 in global CD8 T cells. Third row from left to right: gating of CD45RA and CD27 within CD8 T cells, gating of CCR7 expression in CD8 T cells and gating of CD28 expression in CD8 T cells. Bottom row: gating of CD127 and KLRG1 in CD8 T cells, gating of granzyme B and granzyme K in CD8 T cells and gating of Eomes and T-bet in CD8 T cells. (B) Gating strategy for cytokine producing cells after stimulation. Top row from left to right: gating of lymphocytes, single cells (two panels) and live cells. Second row from left to right: gating of CD8 T cells out of live cells, gating of IFNγ producing cells within CD8 T cells, gating of TNFα producing cells within CD8 T cells and gating of IL-2 producing cells within CD8 T cells.

Supplemental Figure 2 - Differentiation of T cell subsets after stimulation

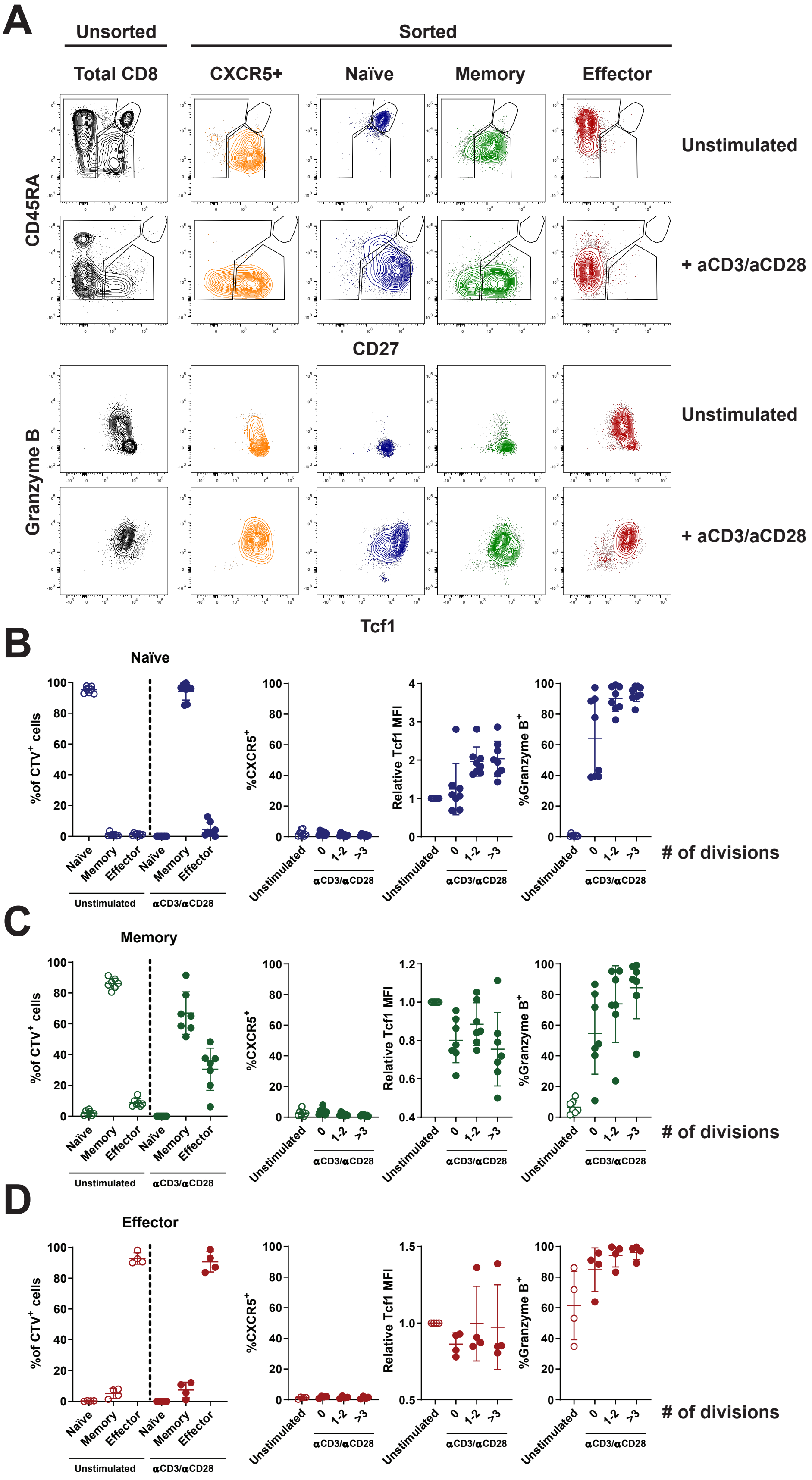

Supplemental Figure 2. Differentiation of T cell subsets after stimulation.

(A) Example of marker expression of unsorted and sorted CD8 T cell subsets before and after stimulation. Top panels show expression of T cell subsets markers CD45RA and CD27. Bottom panels show the expression of memory marker Tcf1 and effector marker granzyme B. (B-D) Differentiation, and expression of CXCR5, Tcf1 and granzyme B of sorted naïve (B), memory (C) and effector (D) CD8 T cells after 5 days of stimulation with anti-CD3/anti-CD28 antibodies (n=7, 3 independent experiments). Data is presented as mean $\pm$ SD.

Supplemental Figure 3 - Subset distribution of CD8 T cells in multiple compartments

A

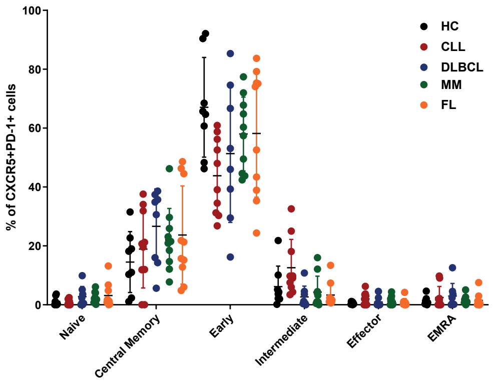

B

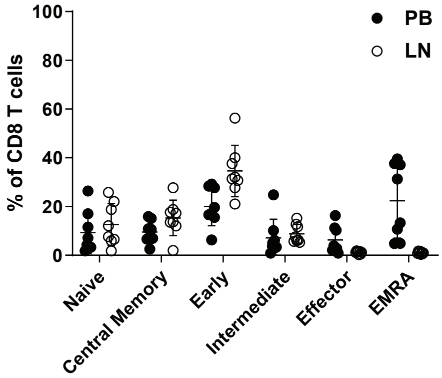

Supplemental Figure 3. Subset distribution of CD8 T cells in multiple compartments.
(A) Subset distribution of CXCR5^{+}PD-1^{+} CD8 T cells in PB of HC (n=8), CLL (n=10), DLBCL (n=8), MM (n=10) and FL (n=10) (10 independent experiments). (B) Subset distribution of total CD8 T cells in HC PB and LN (n=8, 3 independent experiments), showing a lack of effector CD8 T cell populations in LN tissue compared to PB. T cell subsets are defined as naïve (CD45RA^{+}CCR7^{+}CD27^{+}CD28^{+}), central memory (CD45RA^{-}CCR7^{+}CD27^{+}CD28^{+}), early effector memory (CD45RA^{-}CCR7^{-}CD27^{+}CD28^{+}), intermediate effector (CD45RA^{-}CCR7^{-}CD27^{+}CD28^{-}), effector (CD45RA^{-}CCR7^{-}CD27^{-}CD28^{-}) and effector memory expressing RA (EMRA; CD45RA^{+}CCR7^{-}CD27^{-}CD28^{-}). Data are presented as mean $\pm$ SD.
